# A rare case of inflammation after total hip arthroplasty due to a malpositioned prosthesis: A case report

**DOI:** 10.1097/MD.0000000000020468

**Published:** 2020-05-29

**Authors:** Jun Wang, Miao Zhang, Yun Xu, XiaoJing Li, CongCong Wang, XiaoPeng Gao, Meng Xu, XiaoPeng Li, Wei Li, Jun Wang, YiMin Zhang, GuoWei Wang, XueJun Cao

**Affiliations:** a Department of Joint Surgery, Weifang People's Hospital; b Department of Endocrinology and Metabolism, Weifang Municipal Hospital; c Department of Radiology, Changle People's Hospital, Shandong, China.

**Keywords:** infection, inflammation, malposition, total hip arthroplasty

## Abstract

**Rationale:**

Although prosthetic loosening caused by poor prosthesis positioning is common after total hip arthroplasty (THA), an inflammation caused by poor prosthesis positioning is rare. We report a case in which a THA-related inflammation was indeed caused by poor prosthesis positioning.

**Patient concerns:**

A 64-year-old woman was admitted to our hospital with a history of persistent hip pain that had started after she had undergone THA 4 years previously. In addition, she complained of swelling of the hip that had begun 2 months ago.

**Diagnosis:**

Her pain and swelling was initially thought to be caused by an infection, but was eventually diagnosed as inflammation caused by prosthesis loosening, that was in line with finding that her preoperative and intraoperative cultures showed no bacterial or fungal growth. This case posed many questions and difficulties during the diagnostic and treatment stages.

**Interventions:**

Routine diagnosis of periprosthetic suspected infection includes blood test, erythrocyte sedimentation rate, C-reactive protein level, bacterial and fungal cultures, and pathology examinations, which were performed. Finally, this case was eventually diagnosed as inflammation, the prosthesis was removed and antibiotics administered. It was replaced 6 months later.

**Outcomes:**

Except for the erythrocyte sedimentation rate and C-reactive protein levels, X rays, routine blood tests, bacterial and fungal cultures (3 times), and other tests were within the normal range. Positive pathological examinations of synovium during and after the operation indicated chronic inflammation and eliminated inflammation in other areas. Postoperative effect of the second-stage THA was good, with the patient highly satisfied after 6 months.

**Lessons:**

The operative method and position of a joint prosthesis are extremely important. A poorly positioned prosthesis worsens with wear. Wear particles then lead to long-term localized aseptic inflammation with swelling and fever and eventually to low-virulence infection. Prosthetic loosening may be found even at long-term follow-up evaluations after THA in patients with a poorly positioned prosthesis, eventually leading to the need for revision. We had 2 questions: should early revision be considered when a prosthesis had not been properly positioned? In the absence of any confirmation of infection, should a patient suspected of having a periprosthetic infection be treated as early as possible?

## 1 Introduction

Developmental dysplasia of the hip and femoral head necrosis often eventually requires total hip arthroplasty (THA), although its associated complications bring serious problems to patients’ quality of life. Prosthetic loosening and periarticular infection are the most common and most serious complications of artificial joint replacement.^[1–3]^ Loosening and infection around the prosthesis – characterized by local swelling, inconvenient movement, pain, low fever, and the presence of pus – is easily diagnosed. In contrast, an occult infection and inflammation might cause only local pain in the hip joint with unremarkable laboratory examinations, including joint puncture bacterial cultures, making it more difficult to diagnose. Hence, it is difficult to diagnose the origin of aseptic prosthetic loosening.^[4]^

We report a case of hip inflammation caused by a poorly positioned prosthesis during THA. For the patient's safety, we first removed the prosthesis and adopted a conservative treatment plan that included administration of intravenous vancomycin for 6 weeks and oral antibiotics for 6 weeks. Then, after a 6-month waiting period, the patient underwent THA revision surgery. As far as we could determine, this is the first reported case in which an occult infection elsewhere in the body was ruled out, and the diagnosis was a hip inflammation due to aseptic inflammation caused by a poorly positioned prosthesis. We believe that by reporting the clinical characteristics, detailed diagnosis, and treatment of the patient, combined with a review of the literature, we can help provide more early correct diagnoses and further exploration of reasonable treatment.

## 2 Case report

A 64-year-old woman came to our hospital complaining of pain in her left hip that had been particularly aggravated for the past 4 years since she had undergone THA. In addition, she experienced swelling of the hip for past 2 months. The THA had been performed for a left femoral neck fracture. Following the THA, the patient had left hip pain with increased activity, but it was relieved after rest. She therefore did not receive any treatment. Two months before the present admission, the left hip pain worsened along with the appearance of local skin redness and swelling. She came to our hospital and was diagnosed as having a possibly infected, loosening prosthesis following previous left THA.

At admission, the patient was in good condition and had no fever. There was no past history of cancer, kidney disease, human immunodeficiency virus infection, tuberculosis, rheumatoid disease, or hepatitis. The patient denied any history of smoking, drinking, steroid use, or illegal drug abuse. Her family and psychosocial histories were insignificant.

**Figure 1:**
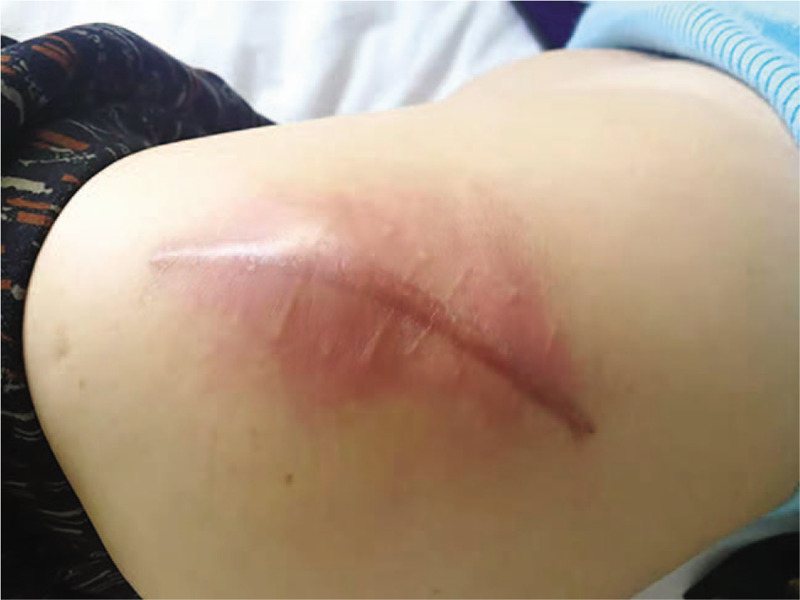
The skin of the hip is red and swollen.

**Figure 2:**
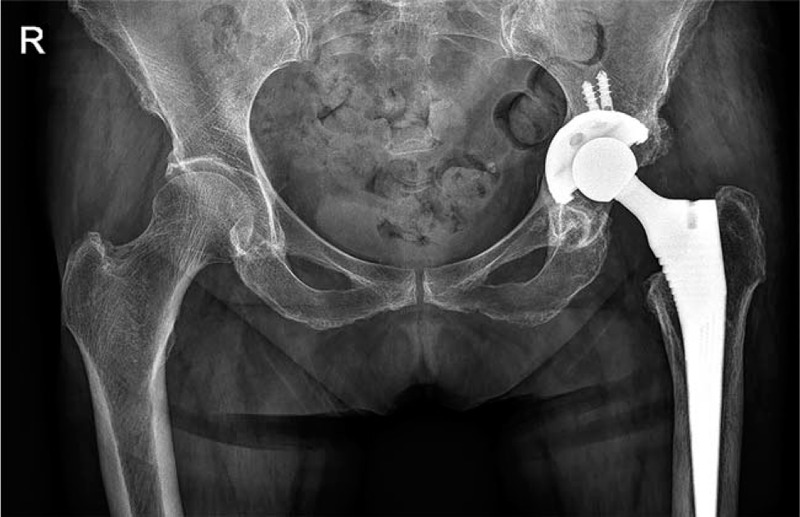
X-ray showed that changes had occurred after the total hip arthroplasty.

Physical examination showed that the left hip joint was swollen, with fluctuant touch and obvious local tenderness (Fig. 1). Plain radiography showed that changes had occurred after the THA (Fig. 2). Color Doppler ultrasonography showed a mixed echo mass and the left hip arthroplasty. No bacteria or fungi were found in 3 puncture-fluid cultures. Laboratory studies revealed the following: white blood cell count 9.29 × 10^9^ (reference normal 4–10 × 10^9^), absolute neutrophil value 4.46 × 10^9^ (2–7 × 10^9^), erythrocyte sedimentation rate (ESR) 97 mm/h (0–15 mm/h), C-reactive protein (CRP) 18.4 mg/L (0–8.0 mg/L).

**Figure 3:**
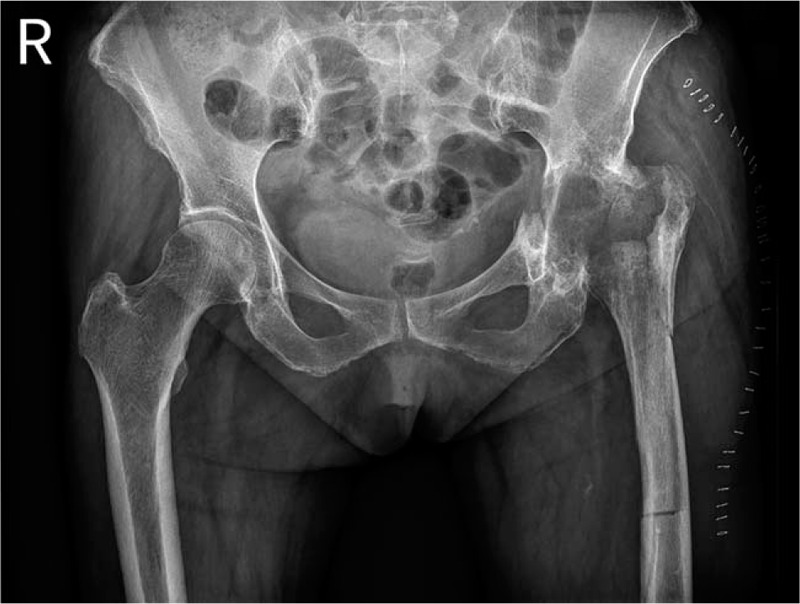
X-ray showed changes after removal of hip prosthesis.

**Figure 4:**
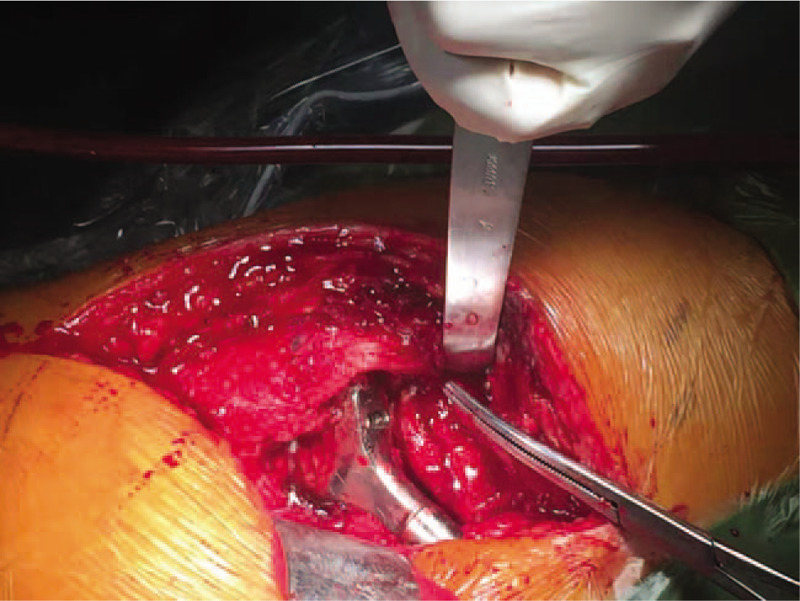
During the operation showed dark blood-reddish fluid.

**Figure 5:**
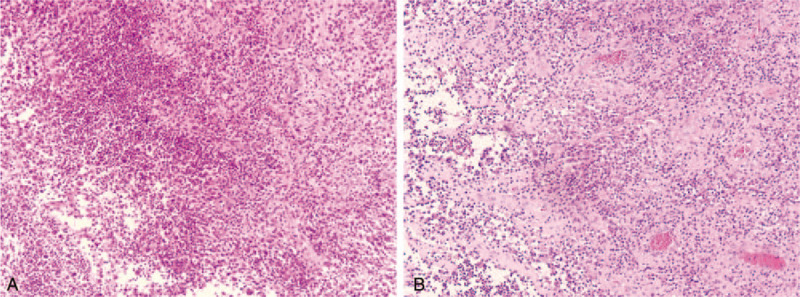
A. Intraoperative rapid freezing pathology suggested chronic inflammation, necrosis, granuloma formation, and small abscess. B. Postoperative pathological results suggested fibrous connective tissue, granulation tissue proliferation, striated muscle, granuloma formation, and suppurative inflammation.

**Figure 6:**
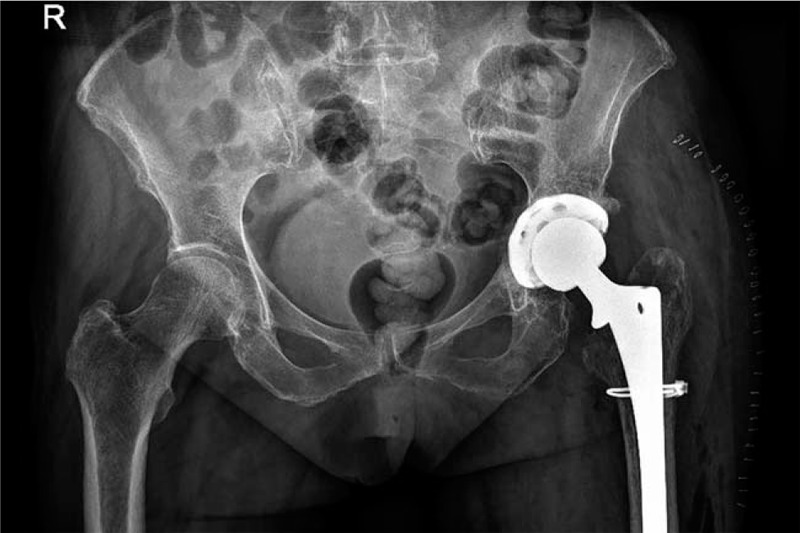
X-ray shows revision after arthroplasty.

Although there were no definitive diagnostic indicators of infection, we still decided to remove the entire prosthesis (Fig. 3). Dark blood-reddish fluid was seen after incising the subcutaneous tissue during the operation (Fig. 4). The prosthesis was loose, and no pus was observed. Tissue culture and pathology evaluation revealed the presence of fibrous connective tissue, granulation tissue proliferation, striated muscle, chronic inflammation, bone, and granuloma formation (Fig. 5). No growth was seen in the bacterial or fungal cultures (i.e., they were aseptic). We treated the patient conservatively with antibiotics. Then, after 6 weeks of intravenous vancomycin and oral antibiotics, her ESR and CRP returned to the normal range, and a waiting period of 6 months, revision THA was performed (Fig. 6). The patient's recovery was uneventful, with good results and without infection.

Ethical review and written informed consent: the Institutional Review Board was waived for this retrospective study. Written informed consent was obtained from the patient for publication of this case report and all accompanying images.

## 3 Discussion

There are many reasons for loosening of artificial hip prostheses, among which the top 3 are the following^[5,6]^:

(1) A biological reaction occurs at the interface of the tissues and the prosthesis due to the wear debris.
(2) Inflammatory prosthesis loosening may be caused by infection after the operation.
(3) The prosthesis was improperly installed, leading to instability, or improper specification and use of the prosthesis, causing it to loosen. In the case described herein, it was considered that the prosthesis was installed in a high, medial position. The patient's daily activities aggravated and wore on the joint, eventually leading to prosthesis loosening caused by aseptic inflammation.

Infection after hip replacement is a serious complication, so it was extremely important to adopt a clear, effective diagnostic method.^[7,8]^ Some occult infections cause symptoms similar to those of aseptic prosthesis loosening (e.g., pain, limited joint movement).^[9]^ There are no obvious radiographic signs of loosening in these patients. At present, a plain radiographic examination and laboratory assessments, such as ESR, CRP, prothrombin consumption time, white blood cell count of synovial fluid, percentage of polymorphonuclear cells, blood and tissue cultures, are the diagnostic methods for recognizing an infection after hip replacement.^[10]^ The combined application of these methods is the basic technique for confirming/excluding the diagnosis of a periprosthetic joint infection (PJI) after THA.^[11,12]^ When these indicators are negative, however, the diagnostic information provided is of relatively limited use.

We initially considered the patient to have a latent infection, but delayed infection after hip arthroplasty usually occurs within 1 to 6 months after surgery, and symptoms such as swelling and pain or sinus formation mainly around the hip joint are rarely reported for more than 6 months postoperatively. According to the accepted diagnostic criteria, PJI (subsequent to index revision) may be diagnosed if at least 1 of the following criteria is present^[4]^:

(1) positive intraoperative cultures on solid medium;
(2) neutrophil count >1760 cells/L and polymorphonuclear cell count >65% in the joint aspirate^[13]^;
(3) presence of a sinus or an abscess.

In our patient, no bacteria or fungi were found in either the preoperative or intraoperative synovial fluid puncture cultures; the routine blood tests and other routine examinations were normal; and the bacterial and fungal cultures (the latter performed 3 times) were within the normal range, so the possibilities that there was a latent infection or an infection somewhere else in the body were completely excluded.

Previous studies had reported that blood laboratory markers, such as ESR and CRP levels, were highly effective in excluding PJI and could be used as first-line screening tests.^[14]^ High ESR and CRP levels may be indications of a latent infection. Their predictive sensitivity is high but poor their specificity.^[15]^ Like other scholars, we deemed that the aseptic inflammation also could lead to the elevated ESR and CRP levels.^[16–18]^ Poor positioning of a prosthesis can cause aseptic inflammation, which, in turn, could lead to increased ESR and CRP levels. Furthermore, aseptic inflammation causes increased effusion, which could induce a low-virulence infection and localized symptoms (i.e., swelling, fever, pain).

## 4 Conclusion

Positioning the prosthesis properly during THA is extremely important. If the prosthesis is not well positioned postoperatively, it increases the “wear and tear” on the hip. The wear particles produced can lead to:

(1) long-term local aseptic inflammation;
(2) local inflammation, swelling, fever; and
(3) low-virulence infection.

In the long term, poor prosthetic positioning can eventually lead to revision. If the prosthesis is not properly installed, early revision should be considered. The “inflammation” in our patient was caused by the poor positioning of the prosthesis. Considering the terrible consequences of infection after arthroplasty, to be safe we treated the patient by removing the damaged prosthesis and administering anti-infective therapy (antibiotics). We later successfully implanted a new prosthesis.

## Acknowledgments

We thank all of the nurses and personnel of Weifang People's Hospital for their cooperation in all the stages of the study.

## Author contributions

**Formal analysis:** XiaoPeng Li.

**Funding acquisition:** YiMin Zhang.
